# Relationship between education and well-being in China

**DOI:** 10.1007/s40847-022-00193-1

**Published:** 2022-09-15

**Authors:** Sijia Liu, Almas Heshmati

**Affiliations:** Jönköping International Business School, Jönköping University, Room B5017, 551 11 Jönköping, Sweden

**Keywords:** Education, Multidimensional well-being, Principal component analysis, Chinese females, C38, C43, D60, H75, I30, J16

## Abstract

Well-being is often quantitatively measured based on individuals’ income or health situation but the relationship between education and well-being has not been fully investigated. It is also important to compare well-being using different individual characteristics especially gender. This paper analyzes well-being using a unique dataset from the Chinese General Social Surveys in 2012, 2013, and 2015. Two measures of well-being are used: self-assessed unidimensional subjective well-being and parametrically estimated multidimensional objective well-being. Objective well-being is a composite parametric index with contributions from different domains of education influenced by identity, capability, and material well-being. These help in understanding the differences between and compare subjective and objective well-being. The results of our descriptive and regression analysis suggests that the multidimensional well-being index differs from subjective well-being in ranking individuals grouped by important common characteristics. These differences are captured by our study which helps to broaden the measurement and analysis of the multidimensionality of the well-being index. Education influences well-being positively, conditional on controlling for identity, capability, material and marital status, and Confucianism. Investments in education and female empowerment which target well-being measures will help reduce the dimensionality of the gender gap in rural China, in particular those attributed to Confucianism.

## Introduction

Chinese women have always had high participation rates in the labor market as compared to other East Asian countries that share the Confucian culture like South Korea and Japan (Chen and Wen 2019; Hare 2016). However, high labor market participation rates do not automatically mean increased well-being. With the one-child policy, educated women’s incomes have increased which makes them more empowered not only in their families but also in society. Women’s empowerment conflicts with the traditional Confucian culture, leading to gaps in well-being among men and women (Chen and Wen 2019; Hannum et al. 2013; Maslak 2011). Improvements in women's education levels lead to changes in their relative incomes which in turn lead to changes in empowerment within families (Chen and Wen 2019). Whether and how these changes affect people’s well-being is an important research question. One can also ask about extent to which education influences women’s well-being in China. How is their well-being different from men’s well-being? This research provides empirical evidence on the relationship between education and well-being from a gender perspective.

Subjective well-being is a personal answer to what individuals feel about their well-being. Most research on economic well-being is related to the relationship between the physical quality of life and subjective well-being (Bertrand et al. 2015; Stutzer 2004). Subjective well-being is also called economic welfare. However, economic well-being slowly adapts into a comprehensive concept which can be viewed as a feeling of fairness or sense of social identity which can then be transformed into a capability that enables people to achieve the functioning capacity that they think is important (Silber 2007). The second measure is parametric and multidimensional which also includes information on identity, capabilities, and material well-being.

There have been rapid social and economic changes in Chinese society through its open door and reform policy and post-reform developments. This has resulted in not only heterogeneity in regional development, but also growing interest in analyzing well-being and the changes in it (Bian et al. 2020; Chen 2018; Cindy and Huang 1998). Economically, at one time China experienced a period in which agriculture was the main source of livelihood. With gradual industrialization, China entered the post-industrial period and today the tertiary industry is playing the main economic role in the country (Canrong 2001; Connelly et al. 2018; Yu and Sarri 1997). As China’s economic transformation progressed, women’s incomes, identity, and values were also influenced leading to changes in their well-being.

Philosophically speaking, gender differences also crept in gradually. According to Fung (1948), the traditional philosophy was far removed from the original Taoism which started around BC 722 according to which nature created females and males for the same reasons so people should follow the principle of nature. The Chinese believed that this would result in a universal phenomenon. Even though the gap between females and males was not large and shifted over time, people still followed the natural principle of living happily (Fung 1949; Harrison 2001). However, the biggest change started in the Song dynasty (starting AC 960), with the ancient Chinese empire expressing an increasing need for political intentions to use the official philosophy of Confucianism. It maintained relations between females and males and believed in man’s superiority with the female’s job being obeying the man. During this time, women’s status deteriorated (Crowell 2005).

From the Song dynasty till the 1900s, people followed Confucianism which resulted in women become their husbands’ appendages. Women did not get a chance to be educated and their feet were banded so that they had no way of escaping (Chen and Wen 2019; Feng et al. 2013; Harrison 2001). This was the identity or value of women in society during the ancient time which is still partially followed (Fung 1948; Ji et al. 2017). A direct result of this is that families prefer to have male children rather than female children because they believe that only the male can inherit the family’s culture or wealth as a female is only an appendage to a male from another family in which she marries.

However, according to Harrison (2001), around the 1900s women’s education became popular. The revolutionary governor’s goal was unbinding women’s feet for the labor force and educating all people for labor quality and women got the first chance to be educated equally with men (Liu 2017). After World War II, the People’s Republic of China emerged as a political system in 1949 and it encouraged people to give birth and fill the gap in labor shortages caused by the war. It also had a slogan: ‘Woman rising as half of the sky,’ which means women had equal power and they had equal rights to work as men (Aitsi-Selmi et al. 2013; Liu 2017). Without denying women’s rights, government and population experts realized that there was a very large and growing population which was making the country short on resources.

Hence, the one-child policy was introduced which led to high abortion rates of girls (Srivastava and Cheema 2019; Summerfield 1997). Female children who survived gained higher education (Liu 2017), while the traditional concept of women’s identity still played an important role (Feng et al. 2013; Shui et al. 2020; Yu and Sarri 1997). Recently, women’s average educational levels have become higher than men’s and the returns on education too are higher for women than for men in urban China (Hannum et al. 2013). Education levels influence incomes which are also attached to empowerment and capabilities which change the hierarchy in a family (Bittman et al. 2003). Thus, there is a natural philosophical conflict between Confucianism and women’s identity and increasing empowerment and capabilities. Confucianism asks women to play an obeying role in the family while increased capabilities enables women to take on more responsibilities in their families and in society.

The purpose of this research is estimating women’s well-being and comparing it with their male counterparts in China. It estimates the relationship between education and well-being accounting for influential factors like marriage and Confucianism. Inclusion of Confucianism in the analysis can have a significant role in associating the sociocultural and religious context in which well-being is influenced by the level of education. It uses two measures of well-being—self-assessed unidimensional subjective well-being and parametrically estimated multidimensional objective well-being. The aim is investigating how education influences well-being. The empirical part estimates and compares the well-being of 34,054 women and men using the Chinese General Social Surveys in 2012, 2013, and 2015. The multidimensional objective well-being index is estimated using a principal component analysis. The different domains of the index include identity, capability, and material well-being. The findings suggest that education influences well-being positively, even conditional on marital status and Confucianism. The multidimensional well-being index differs from subjective well-being in ranking individuals by different characteristics. Marital status has a deep influence on females but not on males’ socioeconomic conditions. Confucianism has negative effects on women’s well-being but not on males’ well-being.

The rest of this study is organized as follows. Section 2 reviews the literature on the relationship between well-being and education. Section 3 describes the data and variables. The method used is presented in Sect. 4. The empirical analysis is discussed in Sect. 5. Section 6 gives the conclusion. The final section gives the implications of the results and the limitations of the study.

## Literature review

### Education and women empowerment

In general, many studies have found a positive relationship between an additional level of education and subjective well-being (Blanchflower and Oswald 2004). According to other studies, middle-level education leads to the highest subjective well-being (Stutzer 2004) but in developing countries like China, education has a more positive impact on well-being than in developed countries because of scarcity of human capital (Aitsi-Selmi et al. 2013; Fahey and Smyth 2004). Education as an indicator of well-being can influence many unobservable traits such as income for women (Hannum et al. 2013), health (Aitsi-Selmi et al. 2013; Dong and Simon 2010), and practice of religious beliefs such as the Confucianism (Frey and Stutzer 2002). According to Graham and Pettinato (2001), once education upgrades a person’s social hierarchy, it influences subjective well-being positively. Thus, education is directly correlated with income, health, hierarchy, and the practice of religion and well-being.

Education levels influence women’s health in China, especially in the low-income quartile as educated women have lower obesity (Aitsi-Selmi et al. 2013), which is evidence of education’s indirect influence on health and well-being. Healthy women have higher chances of having better well-being. However, what we are interested in is education’s influence on women’s well-being which is also related to their empowerment and identity. Deshmukh-Ranadive (2005) suggests that education is highly related to a woman’s economic position, which means ability to work, gaining respect, and reducing gender inequalities. Education makes women get bargaining power in their households. All these aspects increase women’s well-being, but their bargaining power can conflict with Confucianism. Confucianism asks women not to argue but obey while bargaining power is destined to give women the power to argue. Using American household data, Bertrand (2013) and Bertrand et al. (2015) found that women whose relative incomes were higher than men’s, spontaneously did more housework to remedy this gender identification. Thus, self-efficacy, knowledge, and competence can balance Confucian’s influence among highly educated women.

According to Maslak (2011), China has made serious efforts at reducing discrimination against women in particular through education which will increase their well-being. Education has influenced women’s marriages because of its empowerment effect. Women are enabled to share childcare and housework and realize their careers’ productivity. Thus, marriage or family status also influence well-being (Yoshikawa and Kamiya 2019). It is also believed that women who are more educated are less likely to marry men with lower levels of education (Hannum et al. 2013). Lei et al. (2014) found that women who married men with higher educational qualifications had higher well-being than others, but this was only significant for the low-income category or in rural areas. Thus, marital status is a factor that changes women’s well-being. According to Yu (2014) empirical evidence shows that in urban areas Chinese women with higher incomes or education levels who are empowered decrease their housework.

### Identity and economics

Akerlof and Kranton (2010) proposed a new approach for forming norms for different groups which helps explain identity economics. According to their research, norms play a very important role in society. People are happier if they feel that they belong to their own social identity (Shon and Barton-Bellessa 2015). Social identity makes them identify with their social conditions. The extent to which people care about their social identity depends on their identity and the fairness of things (Akerlof and Kranton 2010). Thus, because of wider gender norms during this age of economics, philosophical, and policy changes, we can infer that well-being is also influenced by social identity.

A change in the social identity of modern women in China will not only mean their being ignored in the labor market but also lead to identity confusion, which can lead to a decrease in their well-being. Thus, educated women face an uncertain situation in this society. Education not only brings them higher incomes but also a broader horizon which makes them realize the identity conflict, especially in modern urban China despite returns to education being higher for women than for men (Hannum et al. 2013).

### Income, capabilities, and values

Even though education, empowerment, and social norms can influence well-being, the original indicator—income—shows that when income increases, well-being also increases but with a diminishing rate. However, some studies provide evidence of a reverse causation (Graham et al. 2004). There is also some comparative research which shows the effects of a reference group’s income on well-being. Empirical evidence shows that people are happier if their relative income is higher than the reference group (Ferrer-i-Carbonell 2005). The capability here is the ability to get higher incomes. It is also the ability to achieve functioning for their value (Silber 2007) or having a higher rank in society. As Davis and Dolfsma (2015) suggest, capability also provides a new approach for explaining equality, which is related to hierarchy.

Aspirations of higher incomes also influence well-being. High aspirations and ambitions relate to higher incomes but low well-being (DiTella et al. 2007). A higher income means that a person’s social hierarchy improves. Hierarchy and inequality can also influence people’s well-being. Hagerty (2000) found that inequalities decreased well-being based on a sample of individuals from the USA. However, we can assume that if people have the capability to gain a higher social or family position, their well-being will also increase (Silber 2007). Teschl and Comim (2005) argue that previous research agrees that the ability to adapt to a certain unpleasant environment distorts subjective well-being. They add that capability in the well-being function helps guarantee an objective picture of life satisfaction. They also found that capability can be both a positive and a negative factor for well-being.

How does one define an unpleasant and unfavorable environment? It can involve values, for example, a money-is-all attitude will increase alienation in society (Kim 2014). Using empirical evidence based on data from Korea, the USA, and Sweden, Kim (2014) concluded that a money-is-all attitude decreased well-being by increasing alienation. Thus, values or beliefs also play a very important role in well-being, even when one has a lot of money. Values and beliefs are highly correlated. Helliwell (2003, 2006) found that believing in God was related to higher life satisfaction, but only in the USA and this was not significant in Europe. Stronger religious beliefs also make people value other things such as social relations more than income and employment in UK (Clark and Lelkes 2005). According to Chinese values and beliefs, Confucianism is a kind of religion or society rule, which can be seen as a social norm. Thus, Confucianism is strict and influential (Chen and Wen 2019).

**Fig. 1:**
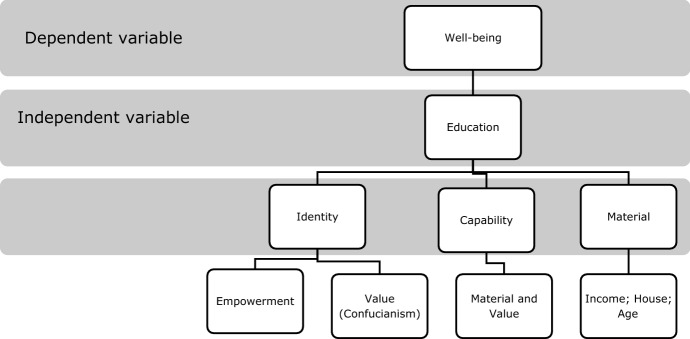
Relationship between well-being and its determinants

There is one special indicator in China—political identity—which not only connects with beliefs but also with values. As an example, if a person is a member of the Communist party, especially women, they get higher incomes and higher life satisfaction in urban areas (Nielsen et al. 2005). Figure 1 shows the specific relationship between the indicators discussed here and well-being.

### More recent holistic research on well-being

Bhagra et al. (2020) analyzed integrated and intergenerational community response in promoting emotional, mental, and holistic well-being during the Covid-19 pandemic. They concluded that the toll on resources, healthcare workers, and the community must be supported through grassroots efforts, in conjunction with efforts at the federal and state levels. Dudovitz et al. (2022) analyzed school-age children's social–emotional well-being and school-related needs during the Covid-19 pandemic among a US sample of parents and school-age children. Their results showed that children had deficits in hyperactivity, peer, and personal areas. Most parents reported school-related needs. Investments in schools are needed to support child health and well-being. Singh et al. (2021) analyzed child well-being in the UK following the Covid-19 lockdowns. Covid-19’s impact on children has been huge, heterogeneous, and mostly indirect. They recommend provision of services and support to meet the children’s needs.

Ni et al. (2020) examined mental illnesses and disorders in the Chinese population because of the pandemic. Mental illnesses lead to disabilities and mental disorders. The Covid-19 pandemic presents a clear threat to mental health globally. They recommend policies that help improve the population’s mental health. Onwumere et al. (2021) examined informal caregivers’ well-being during Covid-19 and observed mood and sleep disturbances. Positive well-being in carers was followed by a more hopeful outlook and fewer symptoms of depression. Couper and Harris (2021) studied the impact of Covid-19 on the well-being of the UK nursing and midwifery workforce during the first wave of the pandemic. They found an adverse psychological impact 3 months after the first wave of the pandemic. These findings tell us how healthcare organizations should respond to their staff’s well-being needs.

Falkingham et al. (2021) examined Chinese middle-aged women’s health and well-being and the associated biosocial correlates during the pandemic. Their aim was unpacking the influence of menopause, lifestyle activities, and social participation. Musculoskeletal health, sleep and memory problems were found to be positively associated with menopausal biological factors among midlife women. Maintaining active social engagements contributed to women’s positive well-being. Wang et al. (2020) estimated the level and changes in well-being in China using CGSS data covering the period 2005–2015. They identified six major functionings. Their results showed that well-being increased by 50% during the studied period. Four functionings (public action, learning ability, protective security and life satisfaction, and economic resources) showed improvements and two functionings (health and shelter) deteriorated. Rural–urban disparity increased particularly in health, shelter, and life satisfaction.

## Data

**Fig. 2:**
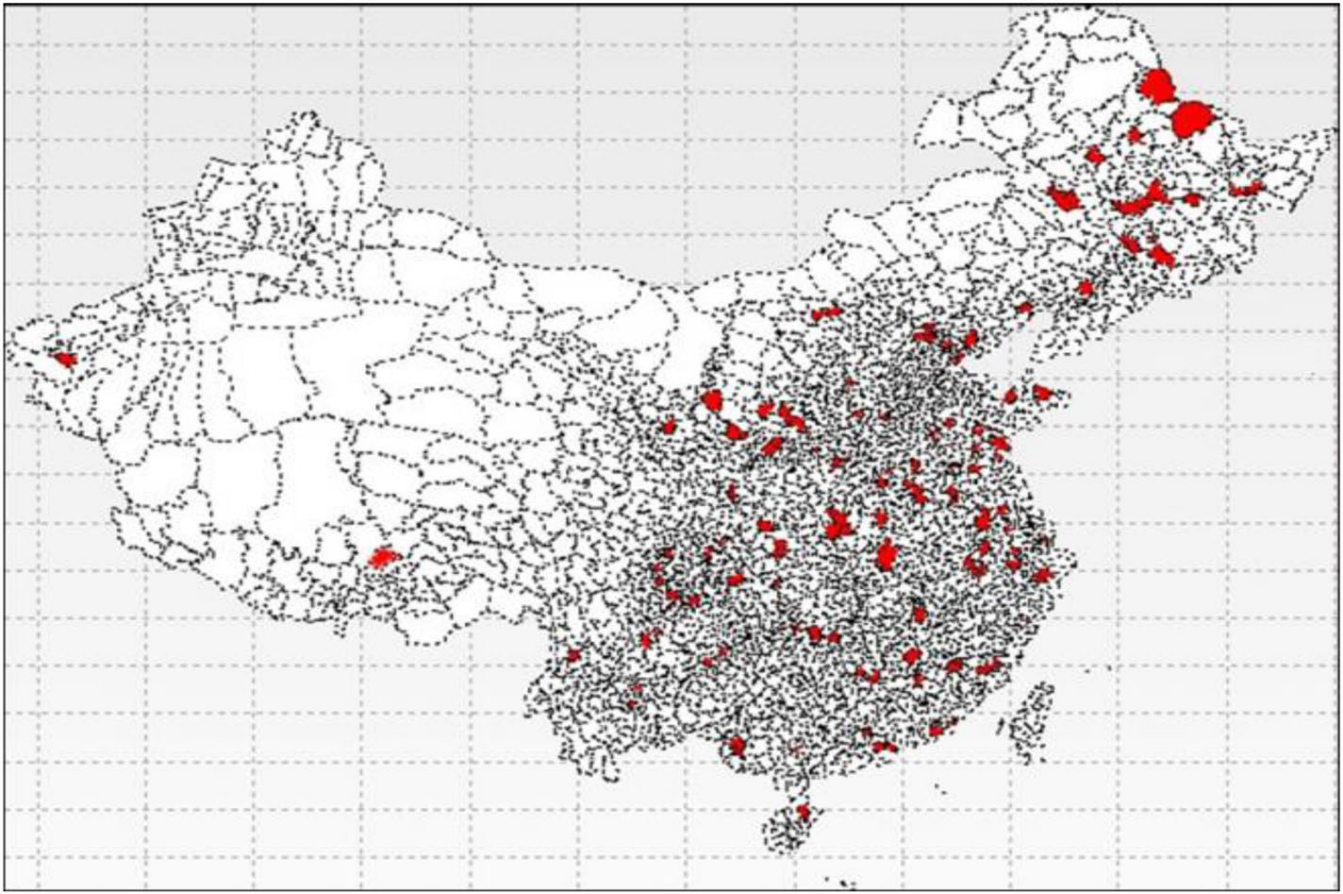
The Chinese General Social Survey (CGSS) data sampling map. The red parts show the sampling locations (CGSS 2020). Source: CGSS (2020). Available at: http://cgss.ruc.edu.cn/index.php?r=index/sample (color figure online)

Data for this study are obtained from the Chinese General Social Surveys (CGSS), which started in 2003 as the first national representative continuous survey aimed at changing relationships in the society’s social structure and people’s well-being. Figure 2 shows how the organization randomly chose people to answer the questionnaire. The red parts in the map show the sampling locations (CGSS 2020). The sample is stratified and as such covers the whole country and weightily crosses provinces, urban and rural parts, and age cohorts. Chen and Wen (2019) used this data for studying the relationship between women and men’s relative incomes and changes in their well-being in families. Wang et al. (2019) also used this dataset to test the spillover effects of identity within a family. They found that political identity influenced well-being. These two studies used data from only one wave. Our study combines data from three waves containing 34,000 observations for increasing the credibility of our results and changes over time.

### Dependent variables

The variables’ definitions are provided in Table 1. The dependent variable is well-being, which can be measured as unidimensional subjective well-being or as a composite multidimensional objective well-being index. Subjective well-being was obtained using a questionnaire asking to what extent individuals felt happy with their lives. The responses were: very unhappy, unhappy, normal, happy, and very happy. Respondents answered the question using their subjective minds. Multidimensional objective well-being is measured as a composite index using a principal component analysis. The index is a combination of all measurable dimensions of well-being. It reflects and increases the objectivity of the measurement.

**Table 1:** Definitions of well-being’s indicators

Variables	Theme	Indicators
Dependent variables	Subjective well-being (SWB)	1 = very unhappy, 2 = unhappy, 3 = normal, 4 = happy, 5 = very happy
	Objective well-being index (OWE)	Calculated by principal component analysis (PCA)
Independent variables	Education level	Education in China is classified as: 1 = illiteracy, 2 = private school for literacy, 3 = primary school, 4 = middle school, 5 = vocational high school, 6 = high school, 7 = technical secondary school, 8 = technical school, 9 = junior college (adult higher education), 10 = college (formal higher education), 11 = university (adult higher education), 12 = university (formal higher education), and 13 = master graduate or doctoral graduate
	Identity: career preference, competition, marriage, quitting work, and sharing housework. Each 5 scale	1 = very disagree, 2 = disagree, 3 = neutral, 4 = agree; 5 = very agree
	Feeling about fairness	1 = totally unfair, 2 = unfair, 3 = neutral, 4 = fair, 5 = totally fair
	Capability: past, present and future and 14 years capability. Each 10 scale	Interval 1–10, 1 is lowest and 10 highest
	Health situation	1 = very unhealthy, 2 = unhealthy, 3 = normal, 4 = healthy, 5 = very healthy
	Income	Specific numbers (in Chinese currency)
	Number of houses	Specific numbers
Essential control variable	Age	In years and cohort group
	Marital status	1 = unmarried, 2 = cohabitation, 3 = first marriage, 4 = remarriage, 5 = separated, 6 = divorced, 7 = widowed
	Gender	1 = male, 2 = female
	Attitude toward Confucianism	1 = good, 2 = neutral, 3 = bad, 4 = very bad
	Province	31 different provinces
	Year	2012, 2013, and 2015

### Independent variables

The most important explanatory variable is education level. Education in China is grade-based, and all the schools and universities are classified into 13 classes following the order from a low to a high level (see Table 1). For example, junior college (adult higher education) is less advanced than a college degree (formal higher education). Adult education is not of high quality like formal education. The system is very different from the western education system, except for the third cycle education. In China, there are three different levels in third cycle education. Class 11 is a university degree (adult higher education), Class 12 is a university degree (formal higher education), and Class 13 is a master or doctoral graduate degree that is equal to the third cycle education in Europe.

The second important control variable is identity that is measured by a female’s empowerment. It is based on 5 questions related to: career, competence, marriage, quitting work, and housework. The answers on a scale of 5 for all the questions cover the extent of agreeing as: very disagree, disagree, do not agree, agree, very agree. The questions are related to differences in career/family priorities, women’s higher competency, better to marry well than doing a job well for women, women should be laid off first when the economy is in recession, and equal sharing of housework among couples.

These 5 questions are the most important questions in traditional Chinese society which capture the state of empowerment in a family, or competition in a workplace between the two genders. Traditional career and competence and quitting work are all about traditional female identity in the workplace and a woman’s competition with males during her career development. Traditional marriage and sharing of housework are about marriage and women’s empowerment within the family. All these directly question a female’s identity conflict in society. They add to the multidimensional well-being index.

The control variables are age, health situation, number of houses that people have, and income, all of which are related to material and health well-being (Heshmati et al. 2015). Most of the variables are categorical but age, number of houses, and income are count or continuous variables. In China, houses are very important not only for the family but also for wealth preservation. House ownership is highly correlated with subjective well-being in urban China (Cheng et al. 2016).

Capability as one of well-being’s indicators is measured based on questions about fairness, hierarchy, and the ability to change the hierarchy at different stages of life—past, present, future, when 14 years old, and the hierarchy at 10 years ago. The response is in 10 categories, ranked from 1 (lowest) to 10 (highest). Capability is a person’s ability to change her/his situation, where questions are related to an individual’s past, present, and future. The hierarchy a person was at when s/he was 14 years old, is important because at that age a person’s world view, philosophy, and values are formed which influence well-being more directly.

We combined the 2012, 2013, and 2015 datasets to get nearly 34,000 observations. Summary statistics of the data is presented in Table 2. Responses to Confucianism form a part of only the 2013 and 2015 surveys with a sample of around 20,000 people. Zero response is due to attrition but also means people’s attitude and is therefore used when comparing individuals. For comparisons the two well-being indices are normalized ranging between zero and 100. The mean subjective well-being is 3.797 (out of 5) and 75.94 (out of 100). While comparing the mean, fairness is only 3.075 out of 5. The mean of education level is also relatively low at 4.87 in the 13 levels of education, which means the average educational category is between middle school and vocational high school. For the identity and capability parts, the ranks 1 to 5 show the extent to which people agree with traditional or new identity questions, and questions on capabilities which are related to hierarchy. The variable Confucianism shows the extent to which people agree with Confucianism (1 very agree and 4 very disagree). The mean 1.93 shows that in general people hold a middle attitude toward Confucianism.

**Table 2:** Descriptive statistics of the data

Variable	Mean	SD	Minimum	Maximum
Normalized subjective well-being (SWB)	75.95	17.27	0	100
Normalized objective well-being (OWB)	48.49	16.26	0	100
Education	4.87	3.07	0	13
Fairness	3.08	1.05	0	5
Health	3.62	1.09	0	5
Income	22,528.28	50,911.26	0	5,000,000
Age	49.29	16.53	17	97
Number of houses	1.10	0.79	0	12
ID career preference	3.42	1.17	0	5
ID competition	2.98	1.19	0	5
ID marriage	3.08	1.17	0	5
ID quitting work	2.15	1.01	0	5
ID sharing housework	3.76	1.03	0	5
Past capability	4.24	1.70	0	10
Present capability	3.48	1.79	0	10
Future capability	5.10	2.21	0	10
14-year-old capability	3.03	1.81	0	10
Confucianism	1.93	1.24	0	4
Province	15.13	8.93	1	31
Gender	1.50	0.50	1	2
Age class	4.25	1.49	1	6
Marital status	3.24	1.41	1	7

The number of observations is 34,043, except for Confucianism. The number of observations with Confucianism is 22,318. Confucianism was not recorded in 2012

## Method

Many studies try to capture well-being using environmental and demographic factors and personal status (Knight and Gunatilaka 2010; Knight et al. 2009). As the SWB (self-assessed subjective well-being) test became popular in recent decades, an increasing number of studies analyzed the relationship between income and SWB (Alesina et al. 2004; Kahneman and Krueger 2006). The questionnaire asks people what they feel about their current well-being. The answer is analyzed using a 5-point scale ranging from very unhappy to very happy. Well-being is analyzed by estimating the least square regression of SWB on women’s educational level conditional on controlling for other variables such as income, age, gender, marital status, and ownership of houses which are objective factors.

SWB is one-dimensional leaving out several dimensions such as education, economic, and housing which are treated as exogenous. A multidimensional index of well-being can be estimated using the principal component analysis (PCA). PCA is commonly applied among others in psychology, education, and development economics. It is an exploratory parametric statistical technique used for reducing the dimensionality of data (Hess and Hess 2018). Reduced dimensionality of the data leads to loss of within dimensional variations but eliminates the problem of collinearity (Heshmati et al. 2015).

PCA is an approximate linear dependency among a set of variables. The relation is written as:

1 $$Y = XB + E$$

where *Y* is a n*p matrix of central variables representing parametric multidimensional well-being; *X* is the n*j matrix of the scores of principal components such as health, housing, values and beliefs, and identity; and *B* is a j*p matrix of coefficients or eigenvectors which are estimated. *E* is a n*p matrix of residuals capturing the unexplained variations by the principal component. *n* is the number of observations, *p* is the number of partial variables, and *j* is the number of variables. Each principal component is a linear combination of the original indicators with coefficients equal to the eigenvectors of the correlation of the covariance matrix. The principal components are sorted according to the declining scale of eigenvalues, which is equal to the variance of the component (Heshmati et al. 2015). The principal component analysis allows adding new components from theories of identity, values, and beliefs to multidimensional well-being.

Chen (2018) used the 2013 CGSS dataset for his study. According to him married women were affected by their personal achievements and characteristics of marriage. Hence, we also account for marital status, gender differences, and education levels. It is also essential to estimate the effects of education on individuals’ well-being where well-being is defined in two different ways (SWB and OWB) and comparing the results. We investigated whether the two measurement methods produced similar or different results. Most of the well-being research on economic aspects focuses on income as the main indicator. However, Akerlof and Kranton (2010) suggest that identity and value should also play a part in well-being. Thus, the two indices of subjective and multidimensional objective well-being are helpful in shedding light on the state of well-being in China.

The last step is an analysis of the relationships between the two measures of well-being and education levels conditioned on different control variables. The models are written as:

2 $$Y_{it} = \alpha_{0} + \sum\nolimits_{J} {\beta_{j} } X_{jit} + \varepsilon_{it}$$

where *Y* is the dependent variable (SWB or OWB); *X* is a matrix of explanatory variables including marital status and Confucianism; *α*_0_ is the intercept; *β* is a vector of unknown parameters to be estimated representing the effects of the determinants of the level of well-being; and ε is the random error term. The subscript *i* refers to individuals and *t* is the period of time. The models are estimated by the least square estimation method with robust standard errors showing the relationship between each well-being and key explanatory variable.

The control variables include age, class, gender, province, year, marital status, and attitude toward Confucianism. Inequalities in resource endowments lead to uneven well-being in different regions or provinces in the country (Chen 2018; Combes et al. 2008). In China, the provinces have partial local autonomy, and they have to follow the central government’s orders. Thus, region of residence expels influence on well-being of the residence. According to Aryee et al. (1999) and Chen (2018), people in mainland China question the importance of marriage and a career, while the well-being of people who live in Hong Kong is highly influenced by the conflict between family and career. Thus, married and unmarried groups have different views and Confucianism is also a gender differential indicator. Confucianism asks women to play an obeying role in their families, but their increasing capabilities induce them to take on more family responsibilities. Men are asked to shoulder all financial responsibilities in the family as per traditional Confucianism without any negotiations (Chen and Wen 2019).

We estimate six different models, Models 1–3 are SWB regressions and Models 4–6 are OWB regressions, where the first model has all the basic control variables along with gender and age. The second is a generalized model with the addition of province and year effects and we add Confucianism to the third model. It should be mentioned here that in case the regression results do not give any conclusive evidence regarding the effects of education on subjective and objective well-being, the mixed results provide a case for exploring latent factors from the results of PCA to find the reasons for the mixed results.

## Descriptive analysis of well-being

### Multidimensional index of well-being

The principal component analysis is used for computing the multidimensional OWB index and its components. The results are presented in Table 3. Part one of Table 3 shows the eigenvalues and the proportion of the variance explained by each component. The eigenvalues exceeding 1 form the well-being index. This index is the weighted average of the first 5 components. The weighted index in comparison to the traditional indices which are based on only the first principal component has the advantage that it utilizes information from different subsets of correlated indicators influencing well-being. The weights of the components are their share of total variance explained by each component (Heshmati et al. 2008, 2015). Together they explain 57.9% of the total variations in well-being. The first component accounts for 20.6% of the variations in the data, while the fifth component accounts for 7.4% of the variations.

**Table 3:** Principal component analysis, eigenvalues, and eigenvectors

Component	Eigenvalue	Difference	Proportion	Cumulative
Comp1	2.878	0.855	0.206	0.206
Comp2	2.023	0.895	0.144	0.350
Comp3	1.128	0.082	0.081	0.431
Comp4	1.046	0.014	0.075	0.505
Comp5	1.033	0.067	0.074	**0.579**
Comp6	0.966	0.041	0.069	0.648

Principal components (eigenvectors)					
Variable	Comp1	Comp2	Comp3	Comp4	Comp5
Income	0.092	− 0.014	**0.415**	**0.437**	− 0.280
Education	**0.310**	− 0.171	**0.439**	0.039	0.029
Health	0.226	− 0.006	**0.386**	0.216	0.073
Houses	0.105	0.057	0.037	**0.563**	− 0.030
Identity career	0.040	0.149	− **0.545**	**0.485**	− 0.047
Identity competition	− 0.180	**0.471**	0.059	0.082	0.134
Identity marriage	− 0.181	**0.497**	0.124	0.010	0.028
Identity quitting work	− 0.156	**0.409**	0.279	− 0.100	0.151
Identity housework	− 0.149	**0.373**	0.129	− 0.050	− 0.246
Fairness	0.050	− 0.030	0.035	0.115	**0.896**
Capability past	**0.456**	0.266	− 0.194	0.010	− 0.012
Capability present	**0.419**	0.209	− 0.105	− 0.268	− 0.060
Capability future	**0.413**	0.200	− 0.129	0.098	0.054
14-year-old	**0.404**	0.119	0.089	− **0.308**	− 0.039

Indicators with eigenvectors above 0.3 are considered the main contributors and are shown in bold

The second part of Table 3 shows the eigenvectors where each indicator’s contribution to the principal components is reported. A positive sign means an increasing and a negative sign a decreasing effect of the indicator on the level of well-being. The size of the eigenvector indicates its strength of effect on well-being. Eigenvector with value exceeding 0.30 is considered a statistically significant contributor to the well-being index. In the first component, the capability indicators are mostly above 0.3 and hence they are considered the main contributors. Component 2 shows the effects of identity indicators, while component 3 reflects on the effects of income, education, and health. Component 4 captures incomes, houses, and careers, while the key contributor to the last component 5 is fairness. All eigenvectors shown in bold in the table have positive effects, where career and 14-year-old’s capabilities are exceptions with a negative sign in the third and fourth principal components. Income, health, houses, fairness, and capabilities are key positive contributors to OWB.

The correlation matrix (Table 4) shows the correlation between each of the well-being indices and their determinants. The correlation between SWB and OWB is around 0.294. The use of many indicators leads to SWB being multidimensional and different from the simpler dimension OWB which explains the low correlation between the two indices. Health situation is also highly correlated with OWB. Identity questions are mostly uncorrelated with each other; they measure different aspects of the traditional or new identity. Present hierarchy representing capabilities is highly correlated with the future and past hierarchies. We have tried to derive and explain the reasons for the mixed results about education’s influence on subjective and objective well-being of individuals. The accounting for multidimensional factors in the objective well-being explains the reasons for the mixed results on the influence of education and the two indices’ differences in explaining variations in well-being.

**Table 4:** Correlation matrix (34,043 observations)

Variables	1	2	3	4	5	6	7	8	9	10	11	12	13	14	15	16	17	18	19	20
1 Subjective well-being	1																			
2 Objective well-being	0.294	1																		
3 Education	0.088	0.428	1																	
4 Fairness	0.271	0.134	− 0.070	1																
5 Health	0.218	0.447	0.270	0.005	1															
6 Income	0.066	0.237	0.252	− 0.000	0.099	1														
7 Age	− 0.010	− 0.270	− 0.450	0.131	− 0.390	− 0.090	1													
8 Number of houses	0.108	0.289	0.065	0.041	0.042	0.115	− 0.010	1												
9 ID career	0.002	0.125	− 0.240	0.076	− 0.060	− 0.050	0.117	0.014	1											
10 ID competition	− 0.020	0.126	− 0.200	0.058	− 0.060	− 0.050	0.121	− 0.001	0.502	1										
11 ID marriage	− 0.030	0.132	− 0.150	− 0.020	− 0.050	− 0.040	0.080	− 0.010	0.339	0.373	1									
12 ID quitting work	− 0.050	0.039	− 0.140	0.049	− 0.050	− 0.020	0.123	− 0.001	0.253	0.332	0.284	1								
13 ID sharing housework	0.077	0.240	0.054	0.000	0.026	− 0.010	− 0.050	0.020	0.004	− 0.040	0.010	− 0.090	1							
14 Past capability	0.273	0.778	0.194	0.150	0.185	0.143	− 0.050	0.120	− 0.020	− 0.020	− 0.040	− 0.020	0.035	1						
15 Present capability	0.145	0.653	0.199	0.073	0.110	0.104	0.025	0.069	− 0.050	− 0.030	− 0.030	− 0.030	0.019	0.624	1					
16 Future capability	0.225	0.711	0.223	0.094	0.227	0.111	− 0.270	0.098	− 0.040	− 0.050	− 0.060	− 0.060	0.050	0.688	0.375	1				
17 14-year-old capability	0.108	0.612	0.301	0.011	0.169	0.114	− 0.200	0.060	− 0.090	− 0.070	− 0.040	− 0.040	0.027	0.440	0.582	0.338	1			
18 Gender	0.024	− 0.010	− 0.120	− 0.001	− 0.070	− 0.120	− 0.010	− 0.010	− 0.040	− 0.030	0.049	− 0.080	0.099	0.024	0.006	0.025	0.034	1		
19 Marital status	− 0.050	− 0.180	− 0.310	0.065	− 0.210	− 0.040	0.504	− 0.030	0.069	0.072	0.056	0.055	− 0.040	− 0.060	0.002	− 0.160	− 0.110	0.129	1	
20 Confucianism	− 0.060	− 0.010	0.035	− 0.050	0.029	− 0.010	− 0.080	− 0.020	− 0.010	− 0.020	− 0.010	0.021	− 0.010	− 0.040	− 0.040	0.043	− 0.010	− 0.030	− 0.030	1

ID indicates identity

The regression results show that present capability has a negative effect on SWB and OBW, which is contrary to past and future hierarchies. Education has a negative relationship with traditional identity, but it has a positive relationship with capability. As predicted, education is also positively correlated with income and health, which represent material well-being.

### Heterogeneity in well-being by characteristics

Table 5 gives a summary of well-being by individual characteristics. In general, mean SWB by different characteristics is much higher compared to OWB. The sample applies to dispersion round mean of different characteristics. Education is classified into 13 different classes in ascending order. SWB and OWB are positive functions of the level of education. However, SWB’s standard errors for all classes of education are higher than OWB’s standard errors. The dispersion is more pronounced for lower education levels. When it comes to fairness, the more fairness people feel, the more SWB they gain (Silber 2007). This is also true for the OWB index, which is gained from PCA. It is important to note that the group which thinks society is totally unfair, has the highest dispersion, which is also consistent with Silber’s (2007) findings. For characteristics of health, people who think they are healthy gain a higher SWB and OWB, which is also consistent with Aitsi-Selmi et al. (2013) and Dong and Simon (2010). People who are in the high-income group also gain higher SWB and OWB as compared to the middle- and low-income groups. It indicates that income still plays a very important role in measuring well-being. However, when it comes to age, the under 20-year-old’s group has the highest SWB. SWB is a decreasing function of age below 60 years. This group has a higher SWB than the working age group. OWB decreases with increasing age even for those over 60 years of age.

**Table 5:** Summary of mean well-being by characteristics

Characteristics	Categories	SWB		OWB	
		Mean	SD	Mean	SD
Education	Illiteracy	73.44	19.73	38.27	14.87
	Private school for literacy	76.15	18.19	42.53	14.52
	Primary school	74.67	18.26	43.78	15.06
	Middle school	76.05	16.68	47.65	14.75
	Vocational high school	75.24	16.55	50.77	13.91
	High school	76.43	17.04	51.75	14.70
	Technical secondary school	78.13	15.24	53.21	14.00
	Technical school	73.54	16.52	56.52	14.31
	Junior college (adult higher educ)	78.25	15.24	57.09	14.78
	College (formal higher education)	78.29	15.17	60.04	13.85
	University (adult higher educ)	78.74	14.99	61.20	14.64
	University (formal higher educ)	79.54	13.88	62.88	14.14
	Master or doctoral graduates	79.29	14.84	66.77	14.63
Fairness	Totally unfair	67.55	23.48	43.81	17.43
	Unfair	71.50	18.10	46.86	16.05
	Neutral	74.56	15.53	48.37	16.03
	Fair	80.02	14.35	50.32	15.84
	Totally fair	87.62	17.71	50.33	17.62
Health	Very unhealthy	65.46	24.47	30.99	15.00
	Unhealthy	70.64	19.58	36.87	14.24
	Normal	74.29	16.83	44.84	14.73
	Healthy	77.04	15.23	51.33	14.33
	Very healthy	80.68	16.21	57.28	14.78
Income	Low income	74.72	18.12	36.84	16.16
	Middle income	78.18	15.18	45.54	15.31
	High income	80.67	14.93	61.35	17.65
Age	Age lower than 20 years	79.15	15.92	55.68	14.07
	20–30 years	77.55	16.09	55.86	15.08
	30–40 years	76.08	16.58	52.18	15.76
	40–50 years	74.59	17.29	48.57	15.79
	50–60 years	74.31	18.01	46.59	16.04
	> 60 years	76.81	17.67	43.50	15.73
Marital status	Unmarried	74.83	17.69	54.38	16.32
	Cohabitation	75.53	17.55	50.80	17.38
	First marriage	76.67	16.64	48.75	15.90
	Remarried	74.48	18.97	47.06	16.71
	Separated	66.08	21.76	41.88	18.07
	Divorced	66.61	20.29	45.72	16.84
	Widowed	73.85	19.62	40.75	15.71
Confucianism	Very good	79.36	16.71	52.64	17.46
	Good	77.37	15.85	50.83	15.44
	Bad	68.77	20.11	42.03	15.54
	Very bad	74.32	16.72	50.11	18.78
Gender	Male	75.65	17.02	48.63	16.38
	Female	76.23	17.51	48.49	16.26

**Fig. 3:**
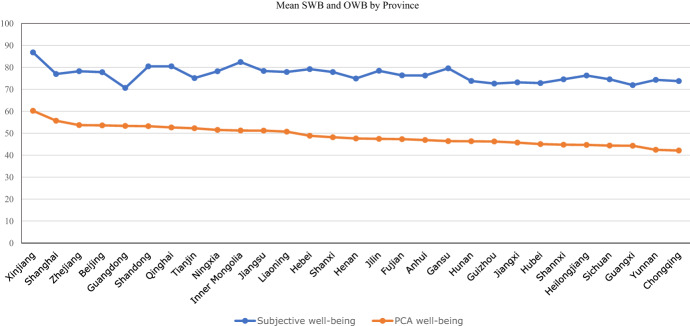
Subjective well-being (SWB) and PCA Objective well-being (OWB) by Province Sorted by level of OWB

Figure 3 ranks SWB and OWB by province in descending order. Xinjiang has the highest OWB and not Beijing, Shanghai, or Guangdong which are normally assumed to be the richest, front runner provinces or cities in China. Xinjiang outperforms in health, fairness, and other aspects considered in OWB. People in Beijing and Shanghai have high OWB but relatively low SWB. Despite its richness, Guangdong has the lowest SWB, while Inner Mongolia and Gansu have low OWB but high SWB.

When it comes to marital status, OWB is different from SWB. Unmarried persons have a higher mean value compared to married persons. However, unmarried males are not happier than married males. Divorce is the worst situation to be in for males according to OWB while for females the worst situation is separation. People who are in their first marriages or are cohabitating feel happier than the remaining groups. Those in their first marriages are the happiest group. The group of separated people has the lowest well-being. Our conclusion is in line with Yu’s (2014) findings.

### Heterogeneity in well-being by confucianism

The paper attempted to ascertain what is intuitively experienced by most people considering the influence of education on the objective and subjective well-being of individuals besides other factors including Confucianism. It is expected that inclusion of Confucianism in the analysis plays a significant role in bringing forth the sociocultural and religious context within which the well-being of the individuals, in particular females, is influenced by education in China.

The most interesting result in Table 5 is that females with a positive and those with a very negative attitude toward Confucianism have relatively higher well-being. The lowest level of well-being is associated with a bad view of Confucianism. According to Chen and Wen (2019), Hannum et al. (2013), and Maslak (2011), Confucianism is a traditional identity. Women who obey Confucian’s rules and are willing to play the role of traditional women feel happier than people who are unaware of their identity and those who have a subtle attitude toward Confucianism and identity. Moreover, women who resist Confucianism and who clearly know that they prefer their new identity and embrace their own choices, have the highest well-being. On the contrary, males with a positive attitude toward Confucianism have the highest well-being. According to Asadullah et al.’s (2018) CGSS results, women, urban residents, and relatively wealthy people are happier in China. More schooling and better health indicate that subjective well-being can be influenced by the objective component; however, after controlled objective factors Confucianism plays an important part in happiness in China.

Lastly, Confucianism’s OWB has characteristics that are similar to its SWB counterpart, that people who agree with Confucianism are happier, while people who disagree with Confucianism are the third happiest group. Thus, Confucianism as the most influential thought in China still leads to identity confusion among women but not among men. However, some women are more willing to resist their traditional identity to gain higher OWB.

The descriptive summary of the two well-being measures, mean and dispersion, does not fully explain all the variables’ relationships. To overcome this limitation, we do a regression analysis between SWB and OWB and their determinants are discussed in the next section to complement the descriptive analysis.

## Regression analysis and a discussion of the results

### The effects of education on well-being

SWB and OWB’s estimation results using the least square method with robust standard errors is presented in Table 6. The regression results show similarities with the descriptive summaries presented earlier. Model 3 shows that SWB is positively correlated with education, health situation, fairness, income, age, number of houses owned, and equally sharing housework, and negatively correlated with quitting a job and present hierarchy. However, SWB is not correlated with traditional marital identity. Conditional on all control variables, the coefficient of the education category 2 is 0.716, which is interpreted as for every higher level of education an average person gains 0.716 units of extra well-being compared with education category 1. SWB is normalized in the range of zero and 100. Thus, a 0.716% increase in well-being is not high compared to the effects of the other indicators of well-being. Higher education has a stronger influence in SWB as compared to OWB. It is worth mentioning here that this result does not give any evidence of stronger effects of education compared to identity, capability, and material well-being.

**Table 6:** Estimation of subjective well-being (SWB) and objective well-being (OWB) indices

	SWB Model 1			SWB Model 2			SWB Model 3			OWB Model 4			OWB Model 5			OWB Model 6		
	Coef.	SE	Sig	Coef	SE	Sig	Coef	SE	Sig	Coef	SE	Sig	Coef	SE	Sig	Coef	SE	Sig
Education 2	1.142	0.234	***	1.126	0.234	***	0.716	0.290	**	3.218	0.047	***	3.097	0.047	***	3.062	0.058	***
Education 3	1.562	0.316	***	1.376	0.318	***	1.262	0.391	***	7.189	0.080	***	6.998	0.081	***	7.015	0.101	***
Education 4	2.502	0.312	***	2.160	0.321	***	1.541	0.395	***	9.657	0.085	***	9.405	0.084	***	9.405	0.105	***
lnIncome	0.096	0.061		− 0.040	0.061		− 0.122	0.074		0.100	0.011	***	0.099	0.012	***	0.069	0.015	***
lnIncome squared	− 0.010	0.004	**	0.005	0.004		0.009	0.005	*	− 0.002	0.001	***	− 0.002	0.001	**	0.001	0.001	
Age	− 0.344	0.036	***	− 0.344	0.036	***	− 0.345	0.044	***	0.053	0.007	***	0.048	0.007	***	0.041	0.009	***
Age squared	0.004	0.000	***	0.004	0.000	***	0.004	0.000	***	− 0.001	0.000	***	− 0.001	0.000	***	− 0.001	0.000	***
Fairness	3.786	0.095	***	3.707	0.096	***	3.610	0.120	***	0.674	0.017	***	0.697	0.018	***	0.711	0.022	***
Health	2.964	0.097	***	2.932	0.097	***	2.987	0.119	***	3.596	0.018	***	3.575	0.018	***	3.581	0.023	***
ID career preference	0.276	0.091	***	0.296	0.090	***	0.289	0.113	**	1.697	0.018	***	1.708	0.018	***	1.722	0.023	***
ID competition	− 0.240	0.090	***	− 0.226	0.089	**	− 0.230	0.111	**	1.465	0.017	***	1.455	0.017	***	1.442	0.022	***
ID marriage	− 0.145	0.085	*	− 0.225	0.085	***	− 0.113	0.107		1.685	0.017	***	1.685	0.017	***	1.701	0.021	***
ID quitting work	− 0.665	0.098	***	− 0.570	0.097	***	− 0.564	0.117	***	0.387	0.019	***	0.403	0.019	***	0.423	0.024	***
ID sharing housework	0.817	0.091	***	0.674	0.091	***	0.747	0.111	***	3.148	0.020	***	3.146	0.020	***	3.137	0.025	***
Past capability	1.541	0.094	***	1.588	0.094	***	1.660	0.115	***	2.846	0.018	***	2.832	0.018	***	2.817	0.023	***
Present capability	− 0.204	0.075	***	− 0.267	0.075	***	− 0.413	0.093	***	1.798	0.015	***	1.780	0.015	***	1.767	0.019	***
Future capability	0.580	0.061	***	0.617	0.061	***	0.476	0.073	***	2.035	0.012	***	2.060	0.013	***	2.054	0.016	***
14-year capability	0.045	0.063		− 0.058	0.063		− 0.080	0.078		1.809	0.013	***	1.781	0.013	***	1.775	0.017	***
Male	0.000			0.000			0.000			0.000			0.000			0.000		
Female	0.734	0.178	***	0.731	0.177	***	0.789	0.217	***	− 0.595	0.036	***	− 0.632	0.036	***	− 0.586	0.045	***
Unmarried	0.000			0.000			0.000			0.000			0.000			0.000		
Cohabitation	2.705	1.020	***	2.762	1.018	***	2.494	1.405	*	0.150	0.207		0.159	0.205		0.446	0.266	*
First marriage	4.410	0.382	***	4.197	0.380	***	3.815	0.457	***	0.448	0.080	***	0.506	0.080	***	0.586	0.099	***
Remarried	3.092	0.757	***	2.563	0.754	***	2.619	0.913	***	0.319	0.141	**	0.441	0.142	***	0.609	0.181	***
Separated	− 2.098	2.070		− 2.156	2.076		− 2.592	2.488		0.990	0.424	**	1.009	0.419	**	0.857	0.460	*
Divorced	− 3.228	0.770	***	− 3.390	0.765	***	− 3.955	0.943	***	− 0.139	0.154		− 0.106	0.154		− 0.081	0.172	
Widowed	1.002	0.524	*	0.930	0.521	*	0.989	0.634		0.039	0.103		0.145	0.103		0.301	0.130	**
Shanghai				0.000			0.000						0.000			0.000		
Yunnan				− 1.602	0.623	**	− 0.808	0.780					− 1.331	0.143	***	− 0.858	0.184	***
Inner Mongolia				4.439	1.099	***	4.279	1.375	***				− 1.537	0.174	***	− 1.400	0.207	***
Beijing				0.156	0.506		0.728	0.630					− 0.762	0.133	***	− 0.605	0.166	***
Jilin				1.750	0.551	***	1.290	0.677	*				− 1.281	0.126	***	− 1.100	0.151	***
Sichuan				− 2.668	0.538	***	− 1.873	0.658	***				− 1.592	0.121	***	− 1.410	0.148	***
Tianjing				− 2.003	0.553	***	− 2.680	0.680	***				− 1.153	0.127	***	− 1.090	0.148	***
Ningxia				1.628	1.184		2.157	1.400					− 2.247	0.183	***	− 1.954	0.226	***
Anhui				− 1.217	0.578	**	− 0.685	0.720					− 1.155	0.133	***	− 0.923	0.164	***
Shandong				1.054	0.521	**	1.106	0.648	*				− 0.848	0.135	***	− 0.738	0.163	***
Shanxi				1.227	0.637	*	0.707	0.799					− 1.130	0.139	***	− 0.940	0.171	***
Guangdong				− 6.958	0.568	***	− 5.977	0.726	***				− 1.447	0.153	***	− 1.005	0.201	***
Guangxi				− 4.047	0.626	***	− 4.362	0.778	***				− 1.180	0.137	***	− 1.015	0.169	***
Xinjiang				4.765	1.813	***	0.000	0.000					− 1.727	0.290	***	0.000	0.000	
Jiangsu				0.419	0.545		− 0.109	0.682					− 0.848	0.133	***	− 0.709	0.159	***
Jiangxi				− 2.968	0.598	***	− 1.417	0.735	*				− 1.228	0.131	***	− 1.088	0.157	***
Hebei				2.089	0.667	***	2.216	0.849	***				− 0.498	0.145	***	− 0.339	0.177	*
Henan				− 3.168	0.536	***	− 2.665	0.665	***				− 1.542	0.116	***	− 1.340	0.139	***
Zhejiang				0.168	0.554		0.656	0.698					− 0.413	0.142	***	− 0.103	0.179	
Hubei				− 3.084	0.546	***	− 3.551	0.684	***				− 1.255	0.120	***	− 0.995	0.146	***
Hunan				− 1.628	0.562	***	− 1.429	0.699	**				− 0.943	0.133	***	− 0.721	0.164	***
Gansu				3.165	0.903	***	2.964	1.105	***				− 1.520	0.149	***	− 1.383	0.177	***
Fujian				0.806	0.656		0.642	0.781					− 0.700	0.164	***	− 0.472	0.207	**
Guizhou				− 3.119	0.754	***	− 1.999	0.870	**				− 1.243	0.167	***	− 0.935	0.198	***
Liaoning				0.631	0.581		0.630	0.741					− 1.099	0.140	***	− 0.879	0.174	***
Chongqing				− 2.013	0.700	***	− 1.603	0.835	*				− 1.670	0.140	***	− 1.383	0.175	***
Shannxi				− 2.180	0.650	***	− 2.036	0.797	**				− 1.764	0.136	***	− 1.641	0.164	***
Qinghai				3.805	1.055	***	2.752	1.281	**				− 1.155	0.219	***	− 1.054	0.282	***
Heilongjiang				0.419	0.563		0.468	0.699					− 1.541	0.125	***	− 1.296	0.149	***
2012b.year				0.000									0.000					
2013.year				− 1.604	0.209	***	0.000						− 0.024	0.040		0.000		
2015.year				0.692	0.208	***	1.923	0.224	***				0.284	0.044	***	0.296	0.05	***
Very agree confuc.							0.000									0.000		
Agree confucianism							− 0.667	0.388	*							− 0.498	0.100	***
Disagree confuc.							− 5.218	0.502	***							− 0.852	0.109	***
Very disagree confuc							− 2.858	1.576	*							− 0.216	0.396	
No attitude							− 1.923	0.461	***							− 0.746	0.106	***
Constant	44.520	1.075	***	46.954	1.161	***	47.935	1.487	***	− 32.411	0.242	***	− 31.075	0.278	***	− 30.655	0.375	***
R-squared adjusted	0.184			0.204			0.212			0.963			0.964			0.963		
Observations	34,043			34,043			22,310			34,043			34,043			22,310		

*F*-test of models	*F*-test result	Test value
Fswb12	M2 better than M1	27.887
Fswb13	M3 better than M1	23.434
Fswb23	M3 better than M2	73.380
Fowb45	M5 better than M4	21.320
Fowb46	M6 not better than M4	− 12.229
Fowb56	M6 not better than M5	− 269.047
Fowb86	M6 better than M8	13.976
Fowb76	M6 better than M7	14.379
Fowb78	M8 better than M7	11.531

Robust standard errors

*^,^**^,^***Indicate significant at the 0.10, 0.05, and 0.01 levels of significance. Confucianism is not observed in 2012. Model 7 and Model 8 are the same as Model 4 and Model 5 but without observations from 2012. Model 6 is a better model than Model 7 and Model 8

The results of the OWB index are very different from the SWB index. The lower level of the former can be explained by the larger dimensions and variations of well-being. The coefficients’ signs show both the positivity and negativity of their effects. However, the signs occasionally change. For example, identity indicators, except traditional career indicators, have a positive relationship with OWB, while in SWB, traditional identity has a negative effect on well-being. Moreover, the capability indicators also have a positive relationship with OWB, while present capability has a negative effect on SWB. Education has a positive effect on OWB. The differences in the signs are attributed to the differences in the dimensions of well-being.

When it comes to the effect of marital status, in SWB’s Model 3 the results show that for males, taking the unmarried group as the reference, the coefficients of the other groups show significant differences. Compared to unmarried males, the first marriage group shows higher well-being, while cohabitation, remarriage, and divorce among females means lower well-being. Marital status influences well-being not only in Lao (Yoshikawa and Kamiya 2019) but also in China. The coefficient of education changes with the conditioning of the other control variables. Different groups have heterogeneous educational effects on well-being. The happiest groups are those practicing cohabitation and those in their first marriages, which is consistent with expected heterogeneity in well-being by marital status.

In OWB, almost all the variables are statistically significant, even the absolute value of the coefficient of education increases as compared to SWB. In all the other groups with different marital status, there is the same significance but with lower coefficient values. This shows that when considering all the information, marital status influencing the well-being and education levels is a positive indicator.

In Model 6, the benchmark of Confucianism agrees with Confucianism. We can see that the coefficient of Confucianism is negative and with a low absolute value as compared to Model 3. People who have a negative attitude toward Confucianism have lower well-being than those who do not disclose how they feel about it. Further, OWB shows different results where almost all the groups have lower well-being than the reference group. After controlling for Confucianism, the coefficient of education does not change much as compared to the SWB model. The only difference between SWB and OWB is information concentration. SWB is a subjective and one-dimensional index, while OWB not only includes material well-being indicators but also identity and capability. Women with a negative attitude to Confucianism have higher OWB, while women have higher subjective well-being when they agree with Confucianism.

It is worth mentioning here that the regression results did not give any conclusive evidence regarding the stronger effects of education on subjective well-being when compared to identity, capability, and material well-being. The mixed results for objective well-being provide a case for exploring the latent factors that emerge from PCA’s results. This may help arrive at the reasons for the mixed results on the influence of education on objective and subjective well-being of individuals in China.

### Gender comparison of well-being

We compare the results of our estimation for males and females. Male SWB is the reference. Females have 0.789 higher subjective well-being, that is, an increase in education level means higher well-being for women as compared to men. What is interesting is that almost all the traditional identity variables are significant for males. A traditional identity is more important for a male’s well-being as compared to a female’s well-being. After controlling for identity, province, and year of observation the regression is credible. Models 1–3 show that after adding control variables, the coefficient for females increases from 0.734 to 0.789. Females and males still have gaps in well-being. OWB shows that women have lower well-being as compared to men. But in Model 6, the coefficient of education for Group 3 also increases while it decreases for the other groups. After controlling for Confucianism, the coefficient of education increases significantly.

### Elasticity of age and income

The regression model is nonlinear in relation to individuals’ income and age as it uses their squares. The total effect computed as derivative of well-being with respect to income and age is estimated and reported in Table 7. Model 1’s elasticity of income shows that a 1% increase in income leads to a − 0.079% decrease in SWB. In Model 3, when year of observation, province, marital status, and Confucianism are controlled for, a 1% increase in income leads to a 0.041% increase in SWB. This pattern is the same with elasticity for OWB Models 4, 5, and 6. A possible reason for the negative elasticity in Models 1 and 2 is that Confucianism has been left out in these models. The elasticity of age in Models 1, 2, and 3 is positive, which means age has a positive relationship with SWB. A possible reason for this is that retirement is a relief for senior citizens. On the contrary, the elasticity of age in Models 4, 5, and 6 is negative because aging is costly and influences health and well-being negatively.

**Table 7:** Mean elasticity of age and Income based on different well-being models

Variable	Mean	SD	Minimum	Maximum
*Subjective well-being (SWB) models*				
Elasticity of income, M1	− 0.079	0.077	− 0.223	0.096
Elasticity of income, M2	− 0.119	0.035	− 0.184	− 0.039
Elasticity of income, M3	0.041	0.072	− 0.121	0.175
Elasticity of income, M4	0.062	0.012	0.040	0.089
Elasticity of income, M5	0.067	0.009	0.051	0.087
Elasticity of income, M6	0.095	0.019	0.053	0.130
*Objective well-being (OWB) models*				
Elasticity of age, M1	0.070	0.139	− 0.201	0.471
Elasticity of age, M2	0.071	0.139	− 0.200	0.472
Elasticity of age, M3	0.071	0.139	− 0.200	0.472
Elasticity of age, M4	− 0.011	0.020	− 0.068	0.027
Elasticity of age, M5	− 0.016	0.020	− 0.073	0.023
Elasticity of age, M6	− 0.013	0.017	− 0.061	0.019

## Summary and conclusion

The main conclusion of this study is that education level is one of the important determinants of individual well-being. The estimation results show that material well-being, identity, and capabilities also contribute to enhanced well-being. Education has a stronger influence on women’s well-being than on men’s well-being. Marital status and Confucianism also influence well-being conditional on different control variables such as gender. However, objective well-being shows that marital status has a bigger influence on females as compared to males. Confucianism has a negative effect on the well-being of females, but not on men’s well-being.

This paper specifically investigated education, identity, and capability’s relationship with well-being by analyzing a unique and large dataset. Most of the existing research does not use these indicators in their models. The use of the principal component analysis provides fruitful information about the dimensions of well-being. When compared to subjective self-assessed unidimensional well-being, the multidimensional objective well-being including material well-being’s information shows different results that are helpful in understanding well-being more objectively.

This study has some policy implications. Investments in higher education are one way of improving well-being in general, especially for females because based on our empirical results education has a stronger effect on females than on males. Women with a higher level of education will have higher well-being, not only in terms of income but will also have a higher chance at own identification and better logic for following their life’s goals. The traditional identity has a negative influence on women’s well-being but is not very important for men. Confucianism also shows similar strong effects on females and lesser effects on males. Thus, investments in education aimed at empowering women must be combined with changes in family laws and regulations related to traditions and ideologies. The government should implement family laws and make people aware of their new identity which includes equality of genders, wages, and empowerment for securing equal rights for women’s inclusive participation in society.

Some practical advice for decision makers is applying equal pay for equal work for the two genders with similar education, age, work experience, and seniority in companies in China. If the law provides protection to women’s career development and basic rights which is currently continuously improving in China, we are confident that data 2 years later will show a totally different picture of well-being. Recently, the Chinese government has put in considerable effort on coming up with a policy for protecting women’s right to maternity leave and encouraging higher birth rates which is a good sign for women’s well-being.

A limitation of this research is that all the variables are categorical, which limits the informative nature of the data. Despite this limitation the number of categories is relatively large which is sufficient for capturing the relationship between education and well-being. A positive aspect of the categorical variables is that unlike continuous variables they allow estimating heterogeneity effects. A second limitation is that we are unable to get the exact relationship between education and identity or capability. Education brings identity conflict because of changes in empowerment and capabilities which are positively influenced by education. Thus, one cannot test their exact relationship but can only combine the information for an estimation of the composite well-being index.

Similar research in the future can be developed in three ways. The first is waiting for the dataset to be updated. Most well-being research focuses on the relationship between income and well-being. This research explores the influence of income, education, and other related variables on well-being, and also does a deeper analysis of the effects of gender and religion dimensions of well-being. Once data for a longer period is available, a multidimensional index of well-being can be constructed by incorporating economic, social, health, housing, living standards, equality, and safety factors using the parametric index. The multidimensional index can then be compared with subjective well-being accounting for individual heterogeneity and assessing the indices’ development over time.

One can also study the relationship between education and other control variables such as identity, capability, and material well-being. This is not easy to investigate because some of the variables are hard to measure or can only be measured in subjective ways. For example, the relationship between education and identity is not linear. Women with higher incomes can do more housework to remedy their traditional identity. Thus, an extension of the model should account for an improved measurement of the variables influencing well-being and their nonlinear and interactive effects. A regression analysis of well-being using panel data should account for issues of endogeneity, simultaneity, interdependence, heterogeneity, and common correlated effects. The use of simultaneous quantile regressions and stochastic dominance will enable a distributional analysis of well-being by different individual and household time-invariant common characteristics and their effects on performance, happiness, and social inclusiveness.

The paper brings richness to a study of well-being by positioning individuals’ well-being as being influenced by education. It contrasts with the schools which have introduced well-being and happiness for the holistic development of students especially during the pandemic. The data used in this study does not cover the Covid-19 pandemic. However, women’s education levels and other characteristics influence their well-being and happiness whether there is a pandemic or not. When data on the pandemic period is available, one can investigate the well-being, happiness, and education outcomes of students and also employees’ productivity.

## Data Availability

Decoded data are available upon request.

## Code availability

Available upon request.
